# A Rare Case of Balo Concentric Sclerosis, a Subtype of Tumefactive Multiple Sclerosis, in a 40-Year-Old Male: Case Report

**DOI:** 10.7759/cureus.24033

**Published:** 2022-04-11

**Authors:** Amro K Al Ashi, Victorien Meray, Adnan M Aziz

**Affiliations:** 1 Translational Research Department, Herbert Wertheim College of Medicine, Florida International University, Miami, USA; 2 Internal Medicine, Jackson Health System, Miami, USA

**Keywords:** cerebrospinal fluid (csf), human papillomavirus (hpv), magnetic resonance imaging (mri), demyelination, multiple sclerosis (ms), tumefactive sclerosis, balo concentric sclerosis (bcs)

## Abstract

Balo concentric sclerosis (BCS) is a rare sub-variant of multiple sclerosis (MS), a demyelinating disease affecting the CNS. BCS is considered a disease of the brain's white matter with a characteristic tumefactive ring signified with alternating myelinated and demyelinated layers, which gives it an "onion-ring" appearance. Our patient is a 40-year-old male with a past medical history (PMH) of human papillomavirus (HPV) who presented to the hospital with acute onset of progressive horizontal diplopia in the left eye and mild right-sided facial weakness and sensation of heaviness in the head. After ruling out stroke, the patient's clinical presentation prompted further investigation with MRI, MR spectroscopy, and an oligoclonal bands' panel. MRI imaging showed a concentric bullseye area of T1 low signal intensity in the left parietal lobe with surrounding edema vasogenic ring enhancement. MR spectroscopy showed a sequence of incomplete ring-enhancing lesions demonstrating a lactate peak and increased choline. The oligoclonal bands' panel, which revealed negative oligoclonal bands, had elevated IgG in the CSF. The patient was diagnosed with BCS based on the clinical presentation, MRI, MR spectroscopy, and oligoclonal bands' panel findings. The patient was started on high doses of methylprednisolone, which improved his symptoms within 24-48 hours of the initial dose.

## Introduction

Balo concentric sclerosis (BCS) is a rare sub-variant of multiple sclerosis (MS) [1-3]. BCS, named after Dr. Jozsef Balo, was first documented and described in 1906 by Marburg [1,4-6]. Historically, diagnosis of BCS was achieved through post-mortem autopsy showing demyelinating lesions [4,6,7]. Nowadays, several diagnostic exams, along with the clinical presentation, are implemented to aid in the diagnosis of this rare disorder, including MRI, CSF analysis, magnetic resonance (MR) spectroscopy, and in some cases, oligoclonal bands panel [1]. Although it is a sub-variant of MS, BCS has distinctive clinical presentation and lesion characteristics seen on MRI, including tumefactive lesion(s) [3]. The term tumefactive is described as MS variants with demyelinating lesions more than 2 cm in size seen on MRI with associated mass effect and edema [4,8]. BCS rings have alternating layers of myelinated and demyelinated neurons. This gives it the characteristic onion-like vasogenic edematous ring(s) with a halo-like appearance most commonly located in the brain's white matter. These findings are very similar in appearance to the ring-enhancing lesions seen in toxoplasmosis [5,8]. Unlike MS, CSF in patients with BCS has a lower frequency of oligoclonal bands, making the diagnosis harder to achieve solely based on CSF analysis. The clinical presentation of BCS varies from MS. BCS is a monophasic and potentially self-limiting episode rather than relapsing episodes of varying degrees of severity of the symptoms seen in MS. For those reasons, many researchers question the consideration of BCS as a sub-variant of MS [3,8].

In the United States, MS affects 400,000 people [9]. Due to the rarity, challenging variation in presentations, and availability of technology to achieve the diagnosis of BCS, accurate incidence rates have not been accurately documented. BCS occurrence is higher in males than females, with a wide range of presenting ages from 4 to 56 years old, with an average age of 36 years old [1,5]. In this case report, we describe the atypical clinical presentation of a 40-year-old male diagnosed with BCS and discuss the uncertainty surrounding the definitive diagnosis and treatment of this condition.

## Case presentation

This case report details a presentation of BCS in a previously healthy 40-year-old Hispanic male. He presented with acute onset of progressive horizontal diplopia in the left eye, mild right-sided facial weakness, and a sensation of heaviness in the head for 48 hours. He reported a blurry vision in the left eye with the onset of diplopia. On further questioning, the patient reported unintentional 30-40 pounds weight loss in the past six months. He endorsed a temporary worsening in the vision with awakening from sleep lasting up to 30 minutes before returning to the level of vision impairment reported at the time of admission. Nothing was able to improve the symptoms. Otherwise, the patient denied having any other symptoms, including fever, headaches, nausea, vomiting, neck pain or stiffness, night sweats, loss of vision, palpitations, shortness of breath, chest pain, abdominal pain, muscle weakness, and sexual dysfunction. He further denied other associated symptoms, including numbness, tingling, history of seizures, family history of neurological diseases, or previous episodes of vision changes. In addition, he also denied a recent history of head trauma, sick contacts, traveling, and past surgeries or hospitalizations. 

On the physical examination, the patient was a well-appearing male of the stated age, well-groomed, and with no signs of distress lying on the hospital bed. The patient had his left eye half-closed because it helped him see better using the right eye only. The patient was hemodynamically stable with a temperature of 36.4 F, blood pressure 112/75 mmHg, respiratory rate of 16 breaths per minute, heart rate of 58 beats per minute, SpO2 97% on room air, and mean arterial pressure (MAP) 99 mmHg. On neurological examination, visual acuity was reduced to 20/25 in the left eye. The findings were consistent with the persistent binocular horizontal diplopia in the left eye towards the lower right and mild generalized right-sided facial weakness that resolved in the following 24 hours. With these symptoms, the patient reported being able to see objects with more clarity within two feet compared to objects further than two feet. Further examination findings included unilateral left optic neuritis. No other focal neurological deficits were observed, including restricted ocular movement or internuclear ophthalmoplegia.

The patient was hospitalized for further evaluation. Brain CT scan showed focal hypoattenuation within the left frontal-parietal white matter with a faint peripherally enhancing rounded 1.8 cm underlining lesion. Stroke was ruled out. Differential diagnoses were infectious vs. neoplastic vs. demyelinating disease.

**Figure 1:**
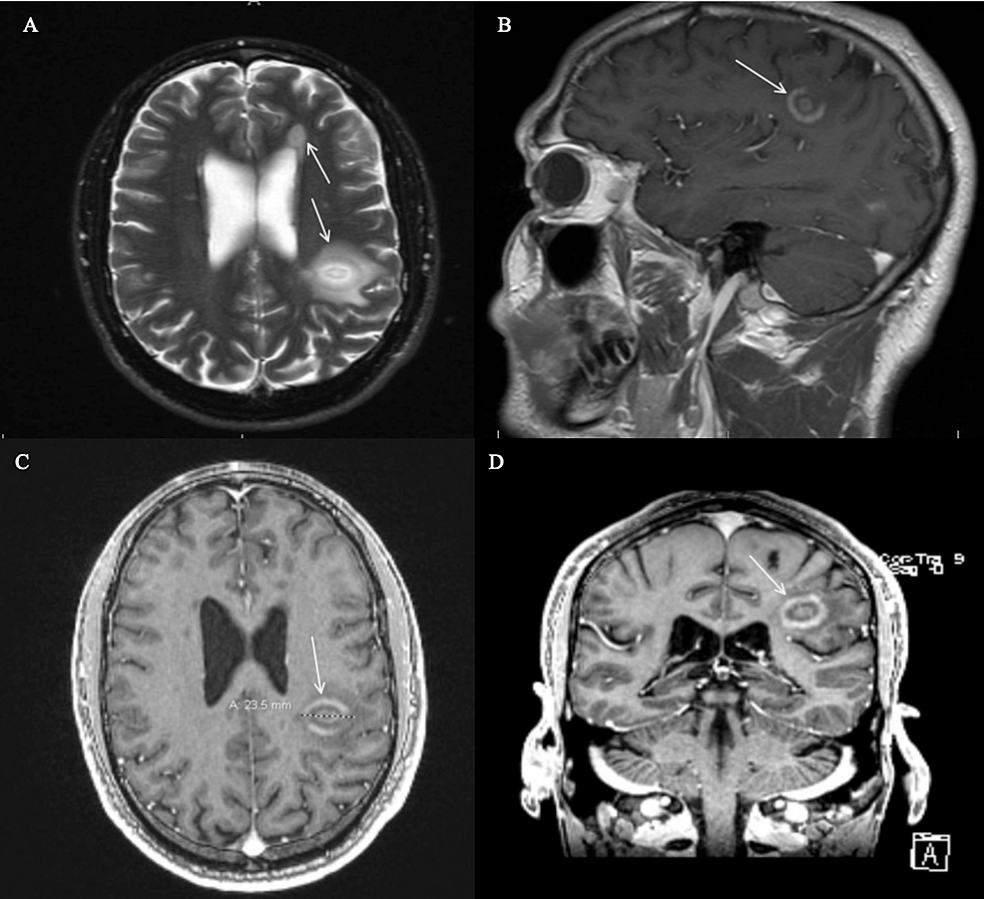
(A-D): MRI brain with and without contrast. Figure 1A shows numerous T2-hyperintense white matter lesions within the subcortical periventricular deep white matter and corpus callosum. Figure 1B shows a concentric bullseye area of T1 isointense and low-signal intensity seen in the left parietal lobe with surrounding vasogenic edema and enhancement measuring 2.1 cm X 1.8 cm X 1.6 cm. Figures 1C and 1D show a smaller enhancing lesion in the left posterior temporal lobe, demonstrating wispy enhancement from an axial and coronal view. Several lesions demonstrate low T1-signal intensity/black holes. There was no intracranial hemorrhage, mass lesion, or acute infarct. There was no extra-axial fluid collection or hydrocephalus.

**Figure 2:**
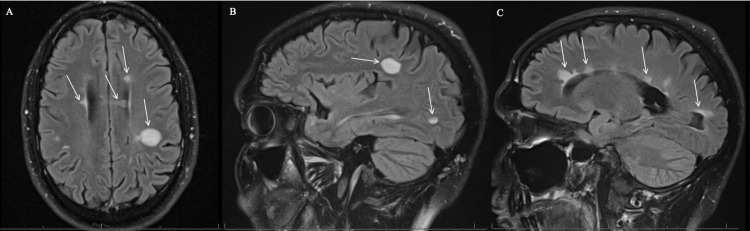
(A-C): MRI brain with and without contrast. Figure 2A showed significantly decreased vasogenic edema associated with the previously described left parietal lobe white matter lesion. Additional supratentorial white matter lesions consistent with MS are again appreciated and not significantly changed. Figures 2B and 2C showed two sagittal views of the decreased vasogenic edema associated with the previously described left parietal lobe white matter lesion. Additional supratentorial and infratentorial white matter lesions consistent with MS are again appreciated and not significantly changed.

To investigate other potential differential diagnoses, an MRI of the brain order revealed numerous T2-hyperintense white matter lesions within the subcortical periventricular deep white matter and corpus callosum with several lesions demonstrating Dawson's fingers (Figure 2C). These findings are consistent with MS. Also, a concentric "bullseye" lesion was identified on T1 MRI in the left parietal lobe with surrounding edema vasogenic ring enhancement measuring 2.1 cm X 1.8 cm X 1.6 cm, which was consistent with BCS superimposed on MS, seen in Figure 1. On MR spectroscopy, a sequence of incomplete ring-enhancing lesions demonstrates lactate peak and increased choline non-specific, which is consistent with BCS. MRI of the cervical spine showed questionable high T2-signal lesions in the spinal cord at the level of C5-C6. These findings prompted conducting an oligoclonal bands panel, which revealed negative oligoclonal bands, elevated IgG of 5.30 mg/dL (vs. normal 0.0-0.7 mg/dL), and elevated protein of 66 mg/dL (vs. normal 15-60 mg/dL) in the CSF.

Progression

The patient's test results were negative for HIV, chlamydia, and toxoplasma. The patient was diagnosed by exclusion with BCS by the neurology, internal medicine, and neurosurgery teams. The patient met all the BCS diagnostic criteria, including painless binocular diplopia and MRI showing a 15 cm ring enhanced lesion and classical tumefactive MS Dawson fingers at the white-gray matter junction [10]. The patient was started on IV methylprednisolone 1g for three days. He had significant improvement in his symptoms with the recovery of almost 90-95% in vision within 24-48 hours. A follow-up CT scan of the head showed decreased edema surrounding the brain lesions on prior imaging. The patient was discharged home with a tapering dose of corticosteroids. He was discharged five days following admission and followed up with neurology after 46 days.

During follow-up, a repeat MRI of the brain and C-spine (Figure 2) showed significant decreased vasogenic edema associated with the previously described left parietal lobe white matter lesion. Additional supratentorial and infratentorial white matter lesions consistent with MS are again appreciated and not significantly changed. MRI of the C-spine showed a high T2-signal lesion in the left dorsal cord of C3 with no acute lesions.

After following up with neurology, the patient would be treated with immunomodulators; however, the patient tested positive for hepatitis B prior to treatment. Although no liver damage was detected, the patient was recommended to follow up with the hepatologist and withhold treatment with immunomodulators until a further clinical assessment is completed.

## Discussion

In the past, BCS was a pathological disease identified and diagnosed post-mortem during an autopsy examination. However, with the improvements and utilization of MRI, this rare subcategory of MS has been better studied and identified, with one of the first MRI-based diagnoses made in 1989 on an antemortem patient [11]. Although there are no definite diagnostic criteria, the diagnosis of BCS is made from a combination of clinical, CSF analysis, and radiological MRI and MR spectroscopy findings. On CSF examination, there is a mononuclear inflammatory infiltration, elevation proteins, and sometimes, oligoclonal bands [12]. An elevation of proteins at 66 mg/mL (normal is up to 45 mg/mL) was noted in this patient during the diagnostic workup.

There is debate on the use of oligoclonal bands in diagnosing BCS. Although there is a strong association between oligoclonal bands and MS, the presence of these immunoglobulins does not help diagnose patients with BCS [13]. One study showed that out of 11 patients with BCS, only one patient tested positive for oligoclonal bands. However, it was discussed that patients with BCS and a positive oligoclonal band screening were at a higher risk of developing MS [14]. Our patient's lab results reveal an oligoclonal antibody, screening negative for oligoclonal bands, and IgG was 5.30 mg/dL (vs. normal 0.0-0.7 mg/dL). The clinical and diagnostic variation in patients with BCS, including the presence or absence of oligoclonal bands, provides more challenges to determine such gold standard treatment and the ability to measure its efficacy. One study performed by Jarius S et al. (2018) found that patients group with a negative oligoclonal band had a higher rate of unfavorable outcomes when compared to oligoclonal band positive groups of patients (20.5 vs. 7.5%). These outcomes consisted of death or no recovery. However, the researchers found no statistical difference in treatment outcomes between both groups [3].

Because BCS can be misdiagnosed and confused with illnesses with similar clinical presentations such as brain abscess, cerebral tuberculoma, sarcoidosis, glioblastoma, primary CNS lymphoma, infarction, or even tumefactive demyelination [1], it is essential to rule out such pathologies prior to arriving at the diagnosis of BCS. The use of MRI to detect concentric-enhancing lesions has highly advanced the diagnosis of BCS. MRI findings in BCS are characterized by unique alternating isointense and hypointense concentric rings on T1-weighted scan [15] and are best visualized using gadolinium dye [16]. They may also be seen on T2-weighted scans consisting of hyperintense lamellae with the rings alternating from hypodense to hyperdense, referred to as an "onion-like appearance." Lesions may be termed "classic lesions," although there is evidence that these rings can be regarded as "complex," ranging from mosaic, rosette, carnation, or even parallel patterns [15]. There is no strict cut-off number for the rings to diagnose a BCS lesion. However, the consensus requires at least two or more concentric demyelinated lesions to call it a "Balo-like lesion" [1]. These findings are consistent with our patient, who had multiple enhanced lesions throughout the brain's white matter detected on MRI. These lesions can also be located in the basal ganglia, pons, and/or cerebellum [1]. In some reported cases, the lesions also affected the optic nerve and spinal cord [14]. In contrast with lesions typically seen with MS, BCS lesions also spare the cortical U-fibers. Current literature descriptions of the MRI findings closely describe the lesions found in our patient's MRI scan, including the multiple lesions in the white matter of the temporal, parietal lobes, and periventricular areas.

Although there have not been any therapeutic trials for BCS due to its rarity and a few patients, the first-line treatment of MS has also been used for cases of BCS, which includes the use of corticosteroids, especially during the active phase of the disease. A few studies have shown that patients typically respond with a five-seven day therapy of 1 g/day of methylprednisolone [12]. Following these guidelines, our patient received the recommended treatment, and within 24 hours, he reported benefiting from this treatment prior to his discharge. Other treatment options were proposed if patients do not benefit from corticosteroids, but those remain controversial with no significant evidence on the efficacy of those treatments. These options include plasma exchange, IV immunoglobulin, cyclophosphamide, and immunosuppression. These treatments provided short-term benefits for some patients. In addition, azathioprine or mitoxantrone have been combined with these treatment modalities to provide long-term treatments [1].

Additionally, there are controversial views regarding maintenance therapy to prevent relapsing Balo-like lesions with no current gold standard treatment. It is recommended that patients be evaluated individually prior to considering such therapy. Drugs such as interferon beta-1a, alemtuzumab, mitoxantrone, natalizumab, and fingolimod have been used with undetermined effectiveness due to varying results [1,17,18]. With the unclear pathophysiology of the disease, different treatment hypotheses were proposed. For instance, Tso AC et al. (2015) proposed that the periventricular demyelination seen in BCS is associated with elevated circulating antiphospholipid antibodies; therefore, treatment with interferon beta-1b may prevent future relapses of BCS. However, these patients did not develop any new lesions on MRI on a one-year follow-up. Therefore, antiphospholipid antibodies' role in demyelination cannot be excluded, and definitive treatment of BCS is still undetermined, requiring more investigation and further assessment for efficacy [19]. 

The prognosis of BCS is thought to be related to the timing of diagnosis. Earlier identification and treatments of Balo lesions have led to a better prognosis [20]. However, as formerly described by Hardy and Miller, prognosis in BCS is related to the severity and clinical presentation, along with BCS lesions manifestation in different disease processes. For instance, the presence of Balo-like lesions in patients with ongoing MS would have a different prognosis than Balo-like lesions manifesting in patients with fulminant Marburg-like disease. Therefore, it is crucial to consider the variability in prognosis when assessing a patient with potential Balo-like lesions. There is no current research evidence supporting BCS affecting the life expectancy of patients with the disease. However, that does not eliminate the potential for BCS to affect the life expectancy of these individuals. Due to its wide range of severity and presentation, BCS patients' variable life expectancy has been associated with the time of diagnosis. Patients diagnosed and treated during earlier stages can live up to 14 years post initial diagnosis [21]. Others, however, have a significantly lower life expectancy and die earlier than others. Capello E and Mancardi GL analyzed the time of death in 17 patients with BCS and found the patients died between five days to eight months following the diagnosis [17]. Thanks to the advancing diagnostic technologies, diagnosis with BCS became more feasible than when it was first documented in 1906.

## Conclusions

Rare cases of BCS are present in typical and atypical ways. Therefore, it is crucial for patients who present with symptoms concurrent with MS to undergo proper workup and imaging to characterize the nature and potential type of MS. Although there is no specific treatment for BCS, proper diagnosis helps communicate the findings with the patient and explain the standard course of the disease. In our patient, follow-up showed that the correct diagnosis and proper treatment benefited the patient, evident by the follow-up MRI reading shows decreased vasogenic edema. From this case report, we hope to raise more awareness about the rare variants of MS, such as BCS, to consider in the differential diagnosis despite its rarity and atypical presentations. Furthermore, we hope our case report adds to the scarce amount of literature available about this rare MS subtype and triggers the need for more research to identify more diagnosis-specific treatments for patients with BCS.

## References

[1] Hardy TA, Miller DH (2014). Balo’s concentric sclerosis. Lancet Neurol.

[2] Balo J (1928). Encephalitis periaxialis concentrica. Arch NeurPsych.

[3] Jarius S, Würthwein C, Behrens JR, Wanner J, Haas J, Paul F, Wildemann B (2018). Baló's concentric sclerosis is immunologically distinct from multiple sclerosis: results from retrospective analysis of almost 150 lumbar punctures. J Neuroinflammation.

[4] Ertuğrul Ö, Çiçekçi E, Tuncer MC, Aluçlu MU (2018). Balo's concentric sclerosis in a patient with spontaneous remission based on magnetic resonance imaging: a case report and review of literature. World J Clin Cases.

[5] Hoang VT, Trinh CT, Van HA (2021). Balo's concentric sclerosis mimicking tumor on magnetic resonance imaging in a young patient. Clin Med Insights Case Rep.

[6] Pakdaman H, Abbasi M, Gharagozli K, Ashrafi F, Delavar Kasmaei H, Amini Harandi A (2018). A randomized double-blind trial of comparative efficacy and safety of Avonex and CinnoVex for treatment of relapsing-remitting multiple sclerosis. Neurol Sci.

[7] Darke M, Bahador FM, Miller DC, Litofsky NS, Ahsan H (2013). Baló's concentric sclerosis: imaging findings and pathological correlation. J Radiol Case Rep.

[8] Lucchinetti CF, Gavrilova RH, Metz I (2008). Clinical and radiographic spectrum of pathologically confirmed tumefactive multiple sclerosis. Brain.

[9] Dilokthornsakul P, Valuck RJ, Nair KV, Corboy JR, Allen RR, Campbell JD (2016). Multiple sclerosis prevalence in the United States commercially insured population. Neurology.

[10] Filippi M, Preziosa P, Meani A (2018). Prediction of a multiple sclerosis diagnosis in patients with clinically isolated syndrome using the 2016 MAGNIMS and 2010 McDonald criteria: a retrospective study. Lancet Neurol.

[11] Airas L, Kurki T, Erjanti H, Marttila RJ (2005). Successful pregnancy of a patient with Balo's concentric sclerosis. Mult Scler J.

[12] Purohit B, Ganewatte E, Schreiner B, Kollias S (2015). Balo's concentric sclerosis with acute presentation and co-existing multiple sclerosis-typical lesions on MRI. Case Rep Neurol.

[13] Kira J (2011). Astrocytopathy in Balo's disease. Mult Scler J.

[14] Simon JH, Kleinschmidt-DeMasters BK (2008). Variants of multiple sclerosis. Neuroimaging Clin N Am.

[15] Charil A, Yousry TA, Rovaris M (2006). MRI and the diagnosis of multiple sclerosis: expanding the concept of "no better explanation". Lancet Neurol.

[16] Amini Harandi A, Esfandani A, Pakdaman H, Abbasi M, Sahraian MA (2018). Balo's concentric sclerosis: an update and comprehensive literature review. Rev Neurosci.

[17] Capello E, Mancardi GL (2004). Marburg type and Balò's concentric sclerosis: rare and acute variants of multiple sclerosis. Neurol Sci.

[18] Ciampi E, Uribe-San-Martin R, Cárcamo C (2020). Efficacy of andrographolide in not active progressive multiple sclerosis: a prospective exploratory double-blind, parallel-group, randomized, placebo-controlled trial. BMC Neurol.

[19] Tso AC, Tsao WL, Chen CY, Yang CF, Peng GS (2015). Combination treatment of interferon β-1b and warfarin for a patient with Baló's concentric sclerosis and antiphospholipid syndrome. Neurologist.

[20] Morrissey M, Ciorciari AJ, Cunningham SJ (2016). A 17-year-old girl with acute onset of hemiparesis. Pediatr Emerg Care.

[21] Wang C, Zhang KN, Wu XM, Gang Huang, Xie XF, Qu XH, Xiong YQ (2008). Balo's disease showing benign clinical course and co-existence with multiple sclerosis-like lesions in Chinese. Mult Scler J.

